# Quantifying protein abundance on single cells using split-pool sequencing on DNA-barcoded antibodies for diagnostic applications

**DOI:** 10.1038/s41598-022-04842-7

**Published:** 2022-01-18

**Authors:** Jenny Sheng, Eldad A. Hod, George Vlad, Alejandro Chavez

**Affiliations:** 1 Integrated Program in Cellular, Molecular, and Biomedical Studies, Columbia University Irving Medical Center, New York, NY 10032 USA; 2 Department of Pathology and Cell Biology, Columbia University Irving Medical Center, New York, NY 10032 USA

**Keywords:** Systems biology, Biomarkers, Biological techniques, Proteomic analysis

## Abstract

Proteins play critical roles across all facets of biology, with their abundance frequently used as markers of cell identity and state. The most popular method for detecting proteins on single cells, flow cytometry, is limited by considerations of fluorescent spectral overlap. While mass cytometry (CyTOF) allows for the detection of upwards of 40 epitopes simultaneously, it requires local access to specialized instrumentation not commonly accessible to many laboratories. To overcome these limitations, we independently developed a method to quantify multiple protein targets on single cells without the need for specialty equipment other than access to widely available next generation sequencing (NGS) services. We demonstrate that this combinatorial indexing method compares favorably to traditional flow-cytometry, and allows over two dozen target proteins to be assayed at a time on single cells. To showcase the potential of the technique, we analyzed peripheral blood and bone marrow aspirates from human clinical samples, and identified pathogenic cellular subsets with high fidelity. The ease of use of this technique makes it a promising technology for high-throughput proteomics and for interrogating complex samples such as those from patients with leukemia.

## Introduction

Cell surface proteins, which make up the surfaceome, have been extensively cataloged for their role in development^1,2^, cell–cell interactions^3^, and signal transduction^4^. Surfaceomes are known to change in disease states and are important for not only disease identification, but also for understanding their biological basis^5,6^ and to identify target therapies^7^. In a clinical context, the ability to distinguish the quantity of important protein markers on patient cells with precision and accuracy is critical for disease diagnosis^8,9^. Furthermore, the importance of cell surface proteins is highlighted by the fact that ~ 60–70% of modern pharmaceuticals target cell surface proteins and over a quarter of human genes code for membrane-associated proteins^10,11^. Though studies have been undertaken to catalog the bulk composition of cell surfaceomes, such population-averaged measurements often fail to detect rare cell types or states which may play meaningful biological roles. Thus, the ability to comprehensively measure the surface proteomes on single cells in heterogeneous populations remains an important goal^12^.

Traditionally flow cytometry has been the method of choice for analyzing proteins present on single cells because of its high throughput capacity and well-benchmarked standards^8,9^. However, flow cytometry has limitations stemming from the spectral overlap of fluorescent conjugates, requiring custom built antibody panels with the ability to interrogate only a limited number of protein targets at a time. This can be particularly problematic in situations where sample cells are scarce, allowing only a limited number of proteins of interest to be examined. Additionally, even in cases where the cell sample is plentiful, relationships between proteins are often incomplete, as not all antigens can be assayed at the same time, limiting studies to only known relationships and potentially missing novel associations predictive of disease outcome or indicative of novel biological processes^12^. Though mass cytometry overcomes some of these barriers, it has not seen wide adoption because of the need for highly specialized equipment^13,14^.

More recently, new techniques have been developed to push the envelope of multiplex protein detection in single cells^15–19^. Many of these techniques seek to leverage the emerging accessibility and sensitivity of next generation sequencing (NGS) for accurate biomolecule (DNA, RNA, protein) detection in single cells. Previous methods aimed at leveraging NGS for detection of proteins on single cells have shown that staining cells with DNA-barcoded antibodies allows for quantitative readouts of protein abundance on single cells using NGS^15–19^. However, a large barrier to adoption of these techniques is the use of proprietary commercial reagents and microfluidics instrumentation necessary for obtaining information from single cells, which adversely affects both cost and accessibility.

Here we present a method for protein detection on single cells using DNA-barcoded antibodies and split-pool sequencing to quantify protein abundances on single cells, similar in concept to the quantum barcoding (QBC) technique published by O’Huallachain and colleagues . Our study was inspired by previous works which used combinatorial split-pool indexing to quantify other cellular properties including RNA, protein, and chromosomal contacts^20–25^. Our implementation of a modified QBC protocol, hereafter named QBC2, employsNGS to sensitively and quantitatively measure dozens of proteins on single cells using reagents, techniques, and equipment familiar and accessible to most molecular biology laboratories. Using peripheral blood or bone marrow aspirates from patient samples, we demonstrate that QBC2 stands up to the diagnostic gold standard of flow cytometry and that it is well suited to characterize complex cellular samples. Our study serves as an important benchmark for future applications of QBC2 to clinical applications. Furthermore, QBC2 is highly scalable, allowing users to simultaneously process multiple patient samples at once, cutting down on reagent usage and expediting processing times. By leveraging NGS and widely accessible molecular biology tools, our method provides a cost-effective strategy for users to perform high-throughput, multiparameter single cell protein analysis, without the need for costly in-house instrumentation.

## Results

### A quantitative and highly modular method for high-dimensional proteomic profiling

**Figure 1:**
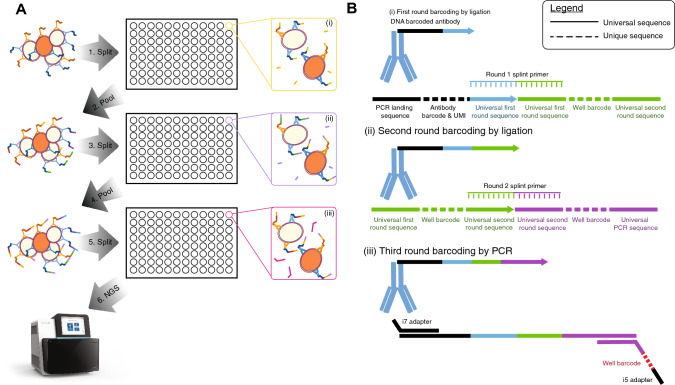
Schematic of QBC2 experimental design. (**A**) Cells are stained collectively with a curated panel of DNA-barcoded antibodies. Stained cells go through a series of split and pool steps where a unique well barcode is appended to the 3′ end of the DNA barcode. A single cell (orange) has been highlighted for clarity as it travels through the barcoding process. (**B**) Schematic of the design of the DNA barcode. First round (i) and second round (ii) well barcodes are appended to the DNA tag that is conjugated to an antibody during the two initial rounds of ligation. During the ligation steps, a splint primer is used to bring the two oligos in proximity to each other and to allow T4 DNA ligase to perform DNA ligation^21^. Third round barcodes (iii) are added through PCR before libraries are prepared for sequencing.

Our implementation of QBC2 leverages a split-pool method to uniquely identify protein expression levels on single cells^20,21,24,26^. The method uses a panel of DNA-barcoded antibodies, which are incubated with a sample of interest, in order to quantify expression levels of proteins on single cells. After washing away unbound antibodies, cells are randomly deposited into a series of wells (Fig. 1A(i)). Within each well, a universal splint primer is used, adapted from Rosenberg et al., to facilitate the ligation of a short DNA oligo containing a unique well barcode to the 3′ end of all DNA-barcoded antibodies within the well (Fig. 1A(ii)^21^). After first round ligation, a short oligo, complementary to the splint primer, is incubated with the cell mixture to block inappropriate ligation during future steps. Subsequently, the cells are pooled, gently mixed, and randomly deposited into a new set of wells, into which a second unique well barcode is added, using a different splint primer. After the second round of ligation, the splint primer is once again blocked and the cells are pooled and deposited into a new plate. In this final plate, PCR is used to amplify and append a third unique well barcode (Fig. 1A(iii)) onto the DNA oligonucleotide coupled to the antibody. The resulting amplicons which are analyzed through NGS each contain an antibody barcode and 3 well barcodes (2 from ligation and 1 from PCR). For each amplicon, the antibody barcode is used to understand the antigen the antibody was bound to and the well barcodes are used to decipher which unique cell the antibody was bound to, as all antibodies interacting with the same cell would be expected to be labeled with the same 3 well barcodes. Collision rates are kept low by ensuring that only a small fraction of the total tripartite well-barcode combinations is used (as barcode doublet rates can be thought of as a Poisson process). By grouping together all antibody sequences with the same trio of well barcodes, a comprehensive profile of the proteins present on each single cell can be determined. For QBC2, experimental capacity is limited only by the number of wells in each round of barcoding and by the desired number of rounds of split-pool.

**Figure 2:**
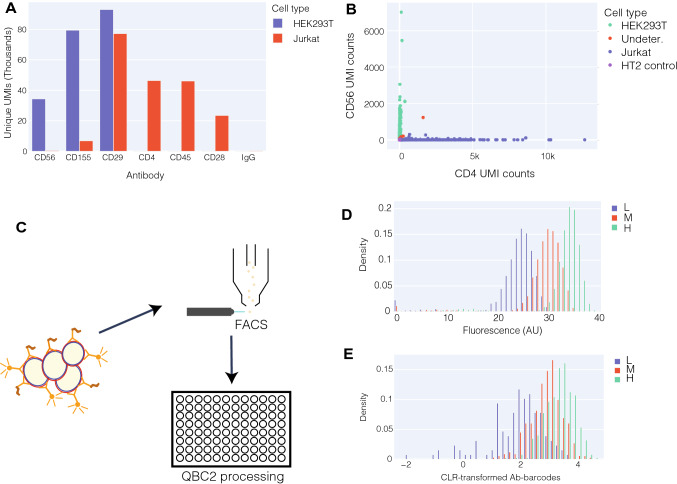
Benchmarking of QBC2 technology. (**A**) HEK293T and Jurkat cells were stained separately with an antibody cocktail containing the antibodies listed. Results from bulk sequencing of DNA-barcoded antibodies bound to stained cells are shown here. (**B**) In a two-cell line mixing experiment where HEK293T and Jurkat cells are used, antibody profiles were used to cluster the cells using k-means clustering (with k = 4) on the top 3 principal components to separate the distinct cell types. As CD56 and CD4 are seen in the bulk measurements to be unique to only one cell type, a visual representation of CD4 and CD56 counts on individual cells shows expression for only one or the other, as identified by clustering (Fig. S2C). HT2 control cells as expected did not stain for either CD56 or CD4. Out of a total of 1262 cells, only 11 were undetermined (0.9%). (**C**) Jurkat cells were co-stained with an antibody mix containing anti-CD4 DNA-barcoded antibody and anti-CD4-FITC. Cells were sorted by FACS for CD4 expression level and high, medium, and low populations were obtained. Sorted cells from the three populations were then uniquely labeled with different hashtag antibodies before collectively passing through QBC2 to quantify CD4 expression through sequencing of anti-CD4 DNA-barcoded antibodies. (**D**) CD4 expression levels are shown as determined by CD4-FITC antibody levels. (**E**) CD4 expression levels are shown as determined by CLR-transformed CD4 antibody-barcode counts.

In order to benchmark QBC2q, we initially performed a mixed cell experiment with two distinct cell lines, Jurkat and HEK293T (Fig. S1). The two cell lines were cultured separately and combined at a 1:1 ratio before staining with a carefully selected panel of antibodies (CD56, CD155, CD29, CD4, CD45, CD28, and isotype control) that were designed to enable us to readily distinguish between the two human cell lines (Fig. 2A). Cells were visualized using the top three components of principal component analysis (PCA) (Fig. S2C, S2D) and clustered into four clusters using k-means clustering. Identity of cell populations determined by clustering were used to visualize Jurkat- and HEK293T-specific markers (Fig. 2B, S2E). Cells of ambiguous provenance were speculated to be from instances of non-specific cell-staining, as identified by their separation along the third principal component (Fig. S2C), which corresponds to an eigenvector predominantly characterized by the presence of IgG and HT3, both markers of non-specific cell labeling.

Jurkat cells spiked in at 5% of the initial pool, stained only with hashtag (HT2, against universally expressed proteins CD298 and β2 microglobulin) DNA-barcoded antibodies, were included in this mixed cell line experiment. These cells were designed to serve as controls, to quantify the rate of barcode swapping during PCR amplification and NGS, along with inappropriate signal caused by antibodies disassociating from their initial target cell and binding a new one during the rounds of split-pooling (Fig. S1). These HT2-stained controls showed no CD56 or CD4 antibody signal (Fig. 2B), suggesting that the signals we observe from QBC2 are from genuine interactions between antibodies and their target cell. As a control for efficient blocking post-ligation, Jurkat cells stained only with HT3 (a hashtag antibody with a different antibody barcode) DNA-barcoded antibodies, were spiked in at 5% of the total cell count after the first round ligation (Fig. S1). These cells were designed to assay the efficiency at which we blocked the first round splint oligonucleotide, before pooling the cells. If the blocking of the round 1 splint oligonucleotide was highly efficient we would expect no HT3-stained cells to be detected in the final analysis, given that they never received the first round of ligation which is required to be a substrate for the second round. As anticipated, no HT3-stained cells (Materials and Methods) were detected in sequencing. These data indicate that barcodes are only ligated within their appropriate well and are not being inappropriately ligated to the incorrect cell during our subsequent rounds of pooling and further manipulation.

To establish the quantitative nature of QBC2, we compared the center-log-ratio (CLR)-transformed scores of antibody counts from QBC2 to the current gold standard for protein quantification, flow cytometry^16,27^. In these experiments, Jurkat cells were co-stained with an antibody cocktail containing both CD4 DNA-barcoded antibodies and CD4-FITC antibodies. Cells were first sorted through fluorescent activated cell sorting (FACS) based on fluorescent CD4-FITC levels. High, medium, and low CD4-FITC cell populations were then stained with hashtag antibodies to mark their FACS sorted population identity before processing via QBC2 (Fig. 2C). CLR-transformed scores of CD4 for each sorted population were compared against FACS measurements to show that QBC2 can quantitatively differentiate protein expression levels in single cells (Fig. 2D,E).

Detailed benchmarking of this method shows that sequencing coverage per cell is lower than traditional single-cell RNA-seq, where 10^5^–10^6^ reads per cell are targeted (Fig. S2)^28^. However, due to the over three orders of magnitude abundance of proteins over RNA, this is expected. Furthermore, for these experiments the lower dimensionality of the sample proteome space (only 5 markers assayed) and the use of unique molecular identifiers (UMIs) on each DNA-barcoded antibody, allows us to accurately identify different cell types (Fig. S2e). Even cells with as few as 100 UMIs detected could be assayed and identified.

### QBC2 can be used to quantify protein expression in complex samples

In cases where multiple samples need to be processed in parallel, DNA-barcoded hashtag antibodies, against universally expressed proteins CD298 and β2 microglobulin, are used to uniquely tag each sample before samples are pooled and stained with a panel of DNA-barcoded antibodies against proteins of interest^29^. In this experiment, we use unique combinations of four hashtag antibodies to uniquely label 10 patient samples (Supp. Table 2). After staining, these cells are then ready for subsequent ligation and PCR steps.

**Figure 3:**
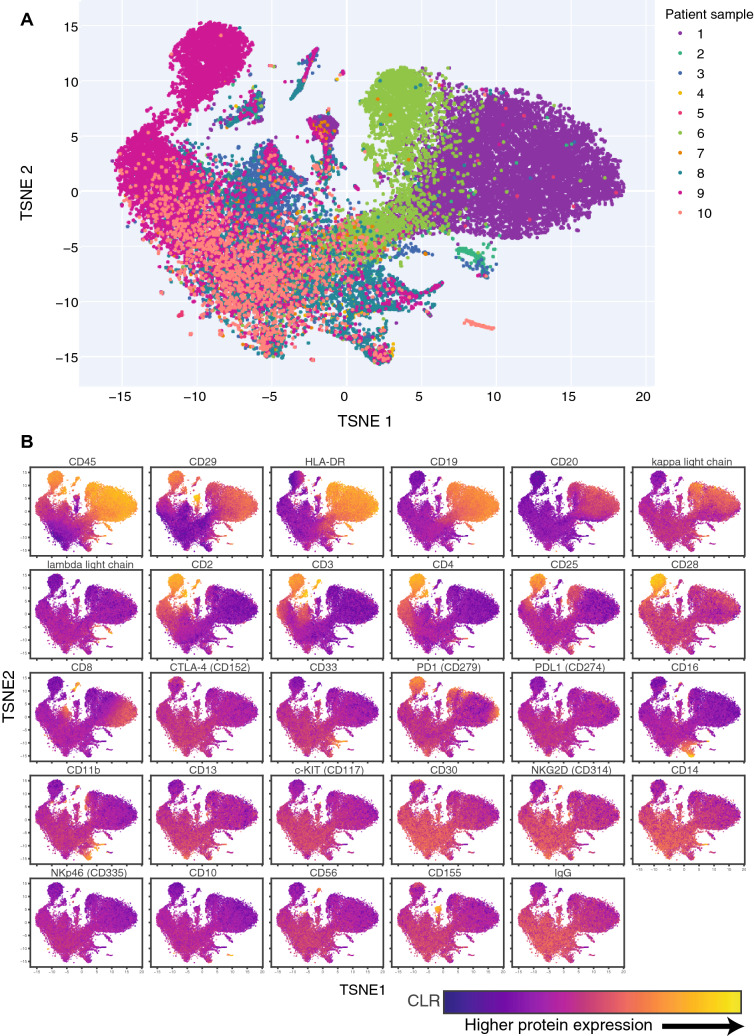
Two dimensional TSNE visualization of pooled patient cell samples. (**A**) Individual cells identified in split pool demultiplexing from 10 pooled patient samples. All cells from a given patient are colored the same. (**B**) Two dimensional TSNE of pooled patient samples, color-coded by CLR-transformed protein scores for all 29 proteins sampled. Color scales are normalized for each individual protein to show maximal dynamic range.

Clinical flow cytometry demands high fidelity due to its critical role in the diagnostic process. We provide initial evidence that not only does QBC2 live up to such data standards, but that it offers advantages by allowing for use of a single antibody panel to simultaneously quantify all antigens of interest, along with multiplexed processing of samples. To demonstrate the potential of QBC2, we conducted QBC2 on 10 varied deidentified patient samples from the Columbia University Immunogenetics and Cellular Immunology Lab. Individual blood and bone marrow samples were uniquely labeled with hashtag antibodies before isolation of peripheral blood mononuclear cells (PBMCs) with Ficoll-Paque reagent. Next, hashtag-labeled PBMCs from all 10 samples were then pooled and collectively stained with a panel of 29 DNA-barcoded antibodies. Cells were taken through two rounds of split-pool ligation with a third round of barcoding performed through PCR. A total of over thirty thousand cells were profiled (Fig. 3).

Visualization of the resulting data in a TSNE plot enabled us to clearly distinguish cells originating from each of the ten patient samples (Fig. S3). Examination of the clustering data reveal the existence of large homogenous groups of cells originating from one or two patients (Fig. 3A). Prominent examples of this are the cell population originating mostly from patient 9 in the upper-left quadrant of the TSNE plot, as well as the population of cells in the upper-right quadrant made mostly of cells from patient 1 and 6 (Fig. 3A). To better understand the nature of these distinct populations, we layered onto the TSNE plot expression data for each of the markers within our panel (Fig. 3B). Examination of the cell surface markers present on the previously mentioned cell populations, revealed them to have patterns of expression associated with hematologic malignancies, explaining their abundance within particular patients and in agreement with the known patient history (Table 1, Fig. S4, and discussed in detail below). Compared to TSNE representations from previous studies that examine single cell protein expression, the representation obtained from QBC2 shows less distinct clusters due to the use of only protein expression data in constructing the representation whereas previous studies have also utilized transcriptional data from RNA-sequencing21,30. Nonetheless, canonical cell types are still clearly distinguishable.

**Table 1:** Patient diagnosis and monitoring information.

Patient	Diagnosis from clinical tests and flow cytometry	Corroborating evidence from QBC2
1	Chronic lymphocytic leukemia (CLL)	Kappa–lambda imbalance in B-cells (kappa expansion)
2	Chronic lymphocytic leukemia (CLL)	Kappa–lambda imbalance in B-cells (lambda expansion)
3	Chronic lymphocytic leukemia (CLL)	Kappa–lambda imbalance in B-cells (lambda expansion)
4	Elevated CD4+ CD25+ (low viability/cellularity)	N/A due to low cellularity
5	Post-transplant lymphoproliferative disorder (PTLD) and non-Hodgkin lymphoma with targeted therapies (low viability/cellularity)	N/A due to low cellularity
6	Chronic lymphocytic leukemia (CLL)	Kappa–lambda imbalance in B-cells (kappa expansion)
7	No pathology (remission monitoring)	Everything within normal range
8	Anemia	Everything within normal range
9	Adult T-cell lymphoma (ATLL)	CD4+:CD8+ imbalance; elevated CD25+, CTLA-4
10	B-cell acute lymphblastic leukemia (B-ALL)	Proliferation of CD19+ CD20−CD45− cells consistent with expansion of pre-B-I cells

Diagnostic information obtained from clinical testing including flow cytometry was compared against evidence obtained from QBC2 to support diagnostic takeaways.

**Figure 4:**
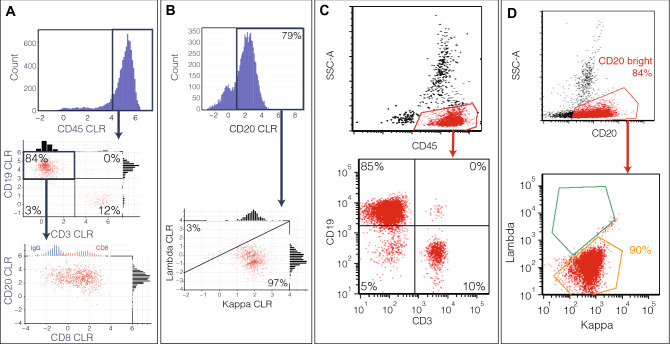
QBC2 CLR-transformed antibody scores and flow cytometry from patient 1 bone marrow sample. (**A**) Cells from patient 1 are gated for CD45+ expression. Further stratification of B-cells in this population shows that CD19+ CD3− gated B-cells show B-cells that have moderate CD8 expression (red) (isotype control expression on CD20+ cells in blue for comparison). (**B**) CD20+ gated B-cells show a pathological expansion of the kappa light chain population. (**C**) Flow cytometry data for the same sample. Monocytes, granulocytes and lymphocytes are identified; lymphocytes are gated for downstream analysis. Population proportion of CD19+ CD3− as identified in flow cytometry similar to QBC2 (85% and 84%, respectively, from panel **A**). (**D**) Bright CD20 expression gated to capture B-cell population. Flow cytometry demonstrates a kappa restricted B cell population, concordant with clonal expansion identified in QBC2.

The information obtained from QBC2 corroborated clinical testing diagnosis, which included but was not limited to flow cytometry (Table 1). Of these, we focus in on two in particular which show intriguing phenotypes to showcase the ability of QBC2 to identify common and pathologic cellular subsets. Analysis of sample 1 focuses on the expanded B-cell population, characterized by CD19+ CD3− (Fig. 4A). A comparison of lambda to kappa light chain ratio indicates an abnormal kappa light chain bias, supportive of the patient history of B-cell chronic lymphocytic leukemia (B-CLL) (Fig. 4B). A normal kappa–lambda ratio ranges from 1 to 2^30^. These signatures and measured expression levels reflect flow cytometry measurements of the same sample (Fig. 4). Interestingly, the T-cell marker CD8, which was not captured in the original flow panel, shows moderate expression on a subset of CD19+ CD20+ cells (Fig. 4A). Though this signature has been reported previously in several cases of CLL, there is little consensus about its prognostic implications^30–32^. Because of the low number of cases that show this phenotype and lack of consensus about its biological implications, diagnostic panels are not designed to pick up this combination of markers. By using a QBC2 setup, rare cell phenotypes such as these can be comprehensively examined without additional effort, to generate new knowledge and drive the field forward.

**Figure 5:**
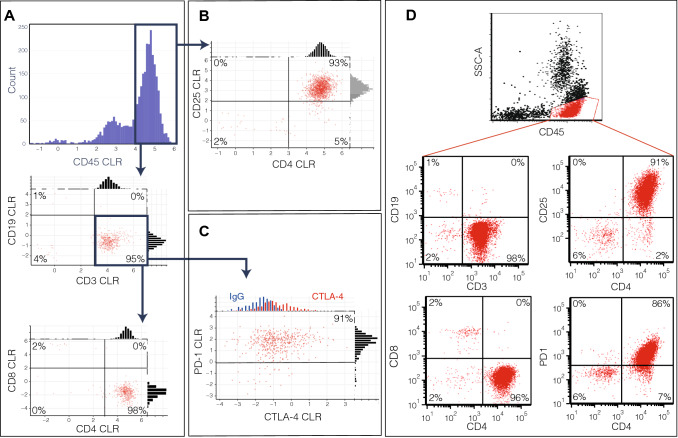
QBC2 CLR-transformed antibody scores and flow cytometry from patient 9 bone marrow sample. (**A**) Cells from patient 9 are gated for CD45+ expression. Gating for T-cells using CD3+ CD19− reveals an expanded CD4+ T-cell population. (**B**) Large proportion of cells revealed CD4+ CD25+ phenotype. (**C**) Population of CD3+ T-cells showed elevated PD-1 and variable CTLA-4 (red) (isotype control in blue for comparison). (**D**) Flow cytometry of the same sample. Cells were gated for lymphocytes, CD45+. Comparison between QBC2 and flow cytometry showed high concordance in gated populations. Population proportions between QBC2 and flow cytometry are consistent throughout.

Scrutiny of sample 9 shows an expanded T-cell population. A comparison of CD3+ CD4+ versus CD3+ CD8+ cells reveals a pathological imbalance between CD4+ and CD8+ T-cells (Fig. 5A). A closer examination of the CD4+ cell population reveals cells with an irregularly elevated level of PD1 and CD25 consistent with the diagnosis of adult T-cell lymphoma (ATLL) originating from peripheral regulatory T-cells (Fig. 5B,C)^33,34^. These signatures are commensurate with the flow cytometric profiles generated for the sample (Fig. 5D). Further analysis of the QBC2 data revealed a subset of ATLL cells with moderate CTLA-4 expression, which is not commonly observed in ATLL and was not assayed with the original flow panel (Fig. 5C)^35^. The significance of elevated CTLA-4 levels within ATLL remains unknown, although in other T cell malignancies increased amounts of CTLA-4 correlates with more advanced disease^36,37^.

## Discussion

QBC2 enables multiplex quantification of expression levels of dozens of cell surface proteins simultaneously on individual cells through a sequencing-based cytometry technique. QBC2 relies on having DNA-barcoded antibodies to label proteins of interest, before the cells are passed through a series of two ligation-based barcoding steps and a final PCR barcoding stage. Multiplexed analysis of samples from parallel experiments is easily accomplished by pre-hashing the samples. Thus, we showed that 10 clinical samples of blood/bone marrow from patients could be multiplexed and analyzed using QBC2.

QBC2 can be scaled to increase the number of cells assayed in two ways; first the number of barcoding wells at each stage can be increased, second the number of barcoding rounds can be expanded (an almost two order of magnitude increase in throughput can be achieved by one additional round of 96-well barcoding). Increasing the number of barcoding rounds requires small changes in primer design but can be seamlessly accommodated in the protocol without significant loss of efficiency. Thus, QBC2 allows for a cost-effective and versatile way to profile dozens of proteins on tens of thousands of cells. As presented, the cost per cell is calculated to be ~ $0.05/cell (Suppl. Table 1). This is driven largely by the cost of sequencing and price of DNA-barcoded antibodies. As sequencing costs have exponentially decreased over the past decade, this technology will likely become increasingly affordable over time. Furthermore, the recent interest in protein detection via DNA-barcoded antibodies has seen an increase in availability of DNA-barcoded antibodies and a reduction in their cost, making it possible to construct evermore diverse panels.

We demonstrate that QBC2 can be used on complex patient samples. Our cell staining workflows require little modification between what is currently used for flow cytometry making it easily adaptable and familiar to most groups. Though studies have previously showcased the potential using a split-pool method to profile protein expression^19,24^, we expand on this principle, demonstrating that it can be used on human clinical material with performance on par with the gold standard clinical diagnostic, flow cytometry. We show that by using a minimal panel of commercially available DNA-barcoded antibodies, we are able to detect both canonical and pathological cell types. By using QBC2, we gain the ability to compare expression levels of multiple proteins directly on the same cell, to gain a more comprehensive understanding of cell state and derive novel biological insight. In future iterations of the technology, we anticipate that QBC2 can be expanded to the detection of intracellular targets using common permeabilization protocols.

While the current ecosystem of single-cell -omics has seen an explosion of development in the sequencing technology space, much of the focus has been on quantifying RNA abundances. Studies have documented that correlations between bulk RNA and protein levels to be modest in many circumstances, with estimates ranging between R ~ 0.4–0.9^38–40^. On the individual cell level, this moderate correlation completely disappears, with evidence showing little to no relationship between RNA and protein levels detected in single cells^16,41^, further cementing the importance of directly probing and assaying protein levels when trying to paint an accurate picture of cell state. QBC2 offers an affordable, rigorous, and quantitative method to perform proteomic profiling with minimal additional equipment or technical expertise outside of that available within most modern molecular biology laboratories, opening the door to easier protein-forward discoveries and diagnostics.

The premise of QBC2 hinges on being able to access and label nucleic acid substrates through ligation, which makes it an easy tool to extend and repurpose. While it may seem that an obvious first expansion of this technique would be to parallelize it with more traditional single-cell RNA sequencing techniques to profile the transcriptome in an unbiased way, previous work points to the low efficiency of split-pool barcoding for unbiased RNA profiling making it a poor candidate for tandem multiplexing with QBC2^42^. A more promising extension of QBC2 would be to selectively capture nucleic acids of interest^13^. To capture RNA, an initial reverse transcription with a targeted capture primer followed by a second strand synthesis would be necessary to form a stable DNA scaffold for the ligation barcoding reactions. Subsequent rounds of barcoding would ligate onto the 3′ end of the synthesized strand. Targeted sequencing would also make it possible to use QBC2 to multiplex CRISPR screening with the quantification of protein abundance.

A long-sought after goal has been T cell antigen discovery, whereby T cell receptors (TCRs) and their subunit pairs can be matched with peptide antigens presented on major histocompatibility complex (MHC) proteins. A comprehensive mapping and association between TCR and antigen pairs could enable us to better understand how to engineer cells to target molecular pathogens or unique tumor antigens. Recent work towards this goal has relied on fluorophore or heavy-metal labeled peptide-MHC (pMHC) ligands presented to libraries of T cells to identify specific TCR-pMHC pairs^43,44^. Instead, by adopting QBC2 techniques, libraries of T-cells can be presented to libraries of DNA-barcoded pMHC ligands to enable high-throughput discovery.

QBC2 is a technique for single cell proteomics that is affordable and scalable. We hope that this technique will provide wider access for the scientific community to pursue complex proteomic studies.

## Materials and methods

### Cell culture and general staining procedure

Jurkat cells (gift from David Ho, Columbia University) were cultured in RPMI media (Gibco) with 10% FBS (Gibco) and 1% Pen Strep (100 × concentrated stock, Invitrogen). HEK293T cells (obtained from ATCC) were cultured in DMEM (Gibco) with 10% FBS and 1% Pen Strep. Cells were maintained in T225 flasks at 37 °C in a humidified atmosphere with 5% CO_2_. HEK293T cells were harvested by gentle trypsinization with 0.05% Trypsin–EDTA (Gibco) for 2 min at room temperature. Cells were counted on a hemocytometer and combined at a 1:1 ratio. A total of 1 million cells were then washed with ice-cold PBS before blocking with 1% BSA and Fc-block at manufacturer’s recommended dilution (BD Biosciences) for 30 min at 4 °C on a rotator.

### Staining of Jurkat and HEK293T cells

All DNA-barcoded antibodies were obtained from Biolegend and are in TotalSeqB format. An antibody cocktail of CD56, CD155, CD29, CD4, CD45, CD28, and isotype control was created by mixing the antibodies at equal ratios. Cells were incubated with a total of 1.5 μg of the antibody mixture per 1 million total cells in a staining volume of 100 μl of blocking solution at 4 °C for 1 h. Stained cells were washed with 1 ml of ice-cold PBS and then centrifuged for 5 min at 300 g. Four more washes were then performed under the same conditions. Finally, stained cells were fixed using 4% PFA for 10 min at room temperature and washed once with ice-cold PBS before depositing them into wells for their first round of well barcode ligation.

### Jurkat CD4 expression cell sorting

Jurkat cells were harvested, counted on a hemocytometer, and resuspended at a density of 1 million cells in 100 μl of blocking buffer. Cells were blocked for 30 min at 4 °C on a rotator. A total of 10 million cells were stained with an antibody cocktail containing several DNA-barcoded antibodies along with anti-CD4 antibodies that were conjugated to either a DNA-barcode or FITC. The antibody cocktail was composed of 1 μg of each of the following DNA-barcoded antibodies, anti-CD4, anti-CD155, and anti-CD29 as well as 2 μg of non-DNA-barcoded anti-CD4-FITC antibody. Cells were incubated with antibodies for 1 h at 4 °C on a rotator and washed 5 times with cold PBS followed by centrifugation. Cells were then sorted based on FITC fluorescence levels into three bins, high, medium and low. After sorting, each population of cells were stained with a hashtag antibody to mark their CD4-FITC expression level. Individual cell populations were counted and resuspended to a density of 1 million cells in 100 μl of blocking buffer before staining with 0.5 μg of the respective hashtag antibodies. Re-stained cells were washed 5 more times to remove unbound antibodies before fixing in 4% PFA in PBS and taken through the ligation barcoding steps.

### Staining of patient samples

Whole blood or bone marrow aspirate from de-identified clinical samples were obtained from the Columbia University Immunogenetics and Cellular Immunology lab (approved by Columbia University, IRB AAAO2000). Approximately 2 ml of either whole blood or bone marrow aspirate from each sample was first stained with 0.5 μg of hashtag antibody for 30 min at 4 °C on a rotator (Supp. Table 2). Peripheral blood mononuclear cells (PBMCs) were then isolated with Ficoll-Paque according to the manufacturer’s protocol. Cells were counted using a hemocytometer and combined stoichiometrically by cell number*—*of note several samples had low cellularity and were not able to be equally sampled. A total of 1 million cells were then blocked with 1% BSA and Fc-block (according to manufacturer’s protocol). Cells were then stained with a total of 4 μg of a panel of antibodies (Supp. Table 3) in 100 μl volume of blocking buffer before being washed five times by centrifugation at 300 g and 4 °C. Stained cells were fixed using 4% PFA for 10 min and washed once before ligation barcoding.

### Split-pool ligation barcoding

To prevent cells from sticking to the sides of the wells, all PCR plates and microcentrifuge tubes used were first blocked with 5% FBS in PBS for at least an hour before use.

Ligation plates were prepared ahead of time and stored until ready to use. Round 1 wells contained 12 μM barcode primer and 11 μM splint primer in water for a total volume of 10 μl. Round 2 wells contained 14 μM barcode primer and 13 μM splint primer in water for a total volume of 10 μl (all primers can be found in Supp. Table 4). Barcoding plates were heated in a thermocycler to 95 °C for 2 min to anneal primers before ramping down to 20 °C at a rate of − 0.1 °C/s and a final hold at 4 °C until ready to use.

Twenty microliters of stained cells were aliquoted into each well of a 96 well plate for the first-round ligation, which contained the round 1 well barcode oligos and round 1 splint primer. The mixture was gently mixed by pipetting up and down several times. Ligation mix containing 1 μl T4 DNA ligase (400,000 units/ml), 5 μl 10 × T4 ligase buffer, and 14 μl water was added to each well. The ligation reaction was performed in a thermocycler at 37 °C for 30 min. The round one splint primer was then blocked to prevent promiscuous ligation upon pooling the wells by the addition of 2.64 μl of 100 μM blocking primer, 2.5 ul of 10 × ligation buffer, and 4.86 μl water to each well. The plate was then incubated in a thermocycler for 30 min at 37 °C to enable the blocking primer to anneal to the splint. Cells were then pooled and mixed by gentle inversion before 100 μl of T4 DNA ligase (400,000 units/ml) was added to the cell mixture and cells were redistributed into 96 round two ligation reaction plates and incubated at 37 °C for 30 min. Round 2 reactions were blocked with termination solution (3.3 μM blocking primer, 0.36 M EDTA; final concentrations).

Cells were pooled after round 2 blocking and counted on a hemocytometer via light microscopy. Cells were diluted to an appropriate final concentration before final round barcoding by PCR. The dilution factor is calculated such that 1 μl of input volume contains enough cells per well such that the total number of cells profiled across all wells does not exceed 2% of the total barcode capacity (to avoid barcode collision doublets). A single round of PCR was performed at 14 cycles. DNA fragments were cleaned up for sequencing through two rounds of bead-based cleanup (at a 0.9 ratio of beads to DNA) according to the manufacturer's protocol (AMPure XP).

### Sequencing, demultiplexing, and bioinformatics

Paired end sequencing was set up to sequence 100 bases from the 5’ end and 50 bases from the 3′ end. Samples were sequenced on NextSeq 500/550 and sequences were demultiplexed in Illumina BaseSpace according to the default quality control settings before subsequent processing.

UMI-tools was used to extract well barcodes from the 5’ read and antibody barcodes along with UMIs from the 3′ read. Count matrices are generated using custom python scripts (Suppl. Table 5). Counts are transformed via center-log-ratio transformation where the CLR scores for cell *x* are defined as

$${\text{CLR}}(x) = \left[ {\frac{{x_{1} }}{g(x)}, \ldots ,\frac{{x_{n} }}{g(x)}} \right]$$

where *g* is the geometric mean of the protein counts for the cell. Dimension reduction is performed via TSNE on the CLR scores using a PCA initialization.

## Supplementary Information

Supplementary Figures.

Supplementary Information 1.

Supplementary Information 2.

Supplementary Legends.

## Data Availability

The sequencing data of experiments are available under NCBI BioProject accession no. PRJNA750440.
