# Supplementary material for: Quantifying protein abundance on single cells using split-pool sequencing on DNA-barcoded antibodies for diagnostic applications
Source: Sci Rep. 2022 Jan 18;12:884. doi: 10.1038/s41598-022-04842-7 (PMC8766443; doi:10.1038/s41598-022-04842-7)
Supplement: Supplementary file 1 — Supplementary Figures. [file 41598_2022_4842_MOESM1_ESM.pdf]

Quantifying protein abundance on single cells using splits-pool sequencing on DNA-barcoded antibodies for diagnostic applications

Jenny Sheng[1,2], Eldad A. Hod[2], George Vlad[2], Alejandro Chavez[2,*]

[1] Integrated Program in Cellular, Molecular, and Biomedical Studies, Columbia University Irving Medical Center, New York, NY, 10032, USA
[2] Department of Pathology and Cell Biology, Columbia University Irving Medical Center, New York, NY, 10032, USA
*Corresponding author

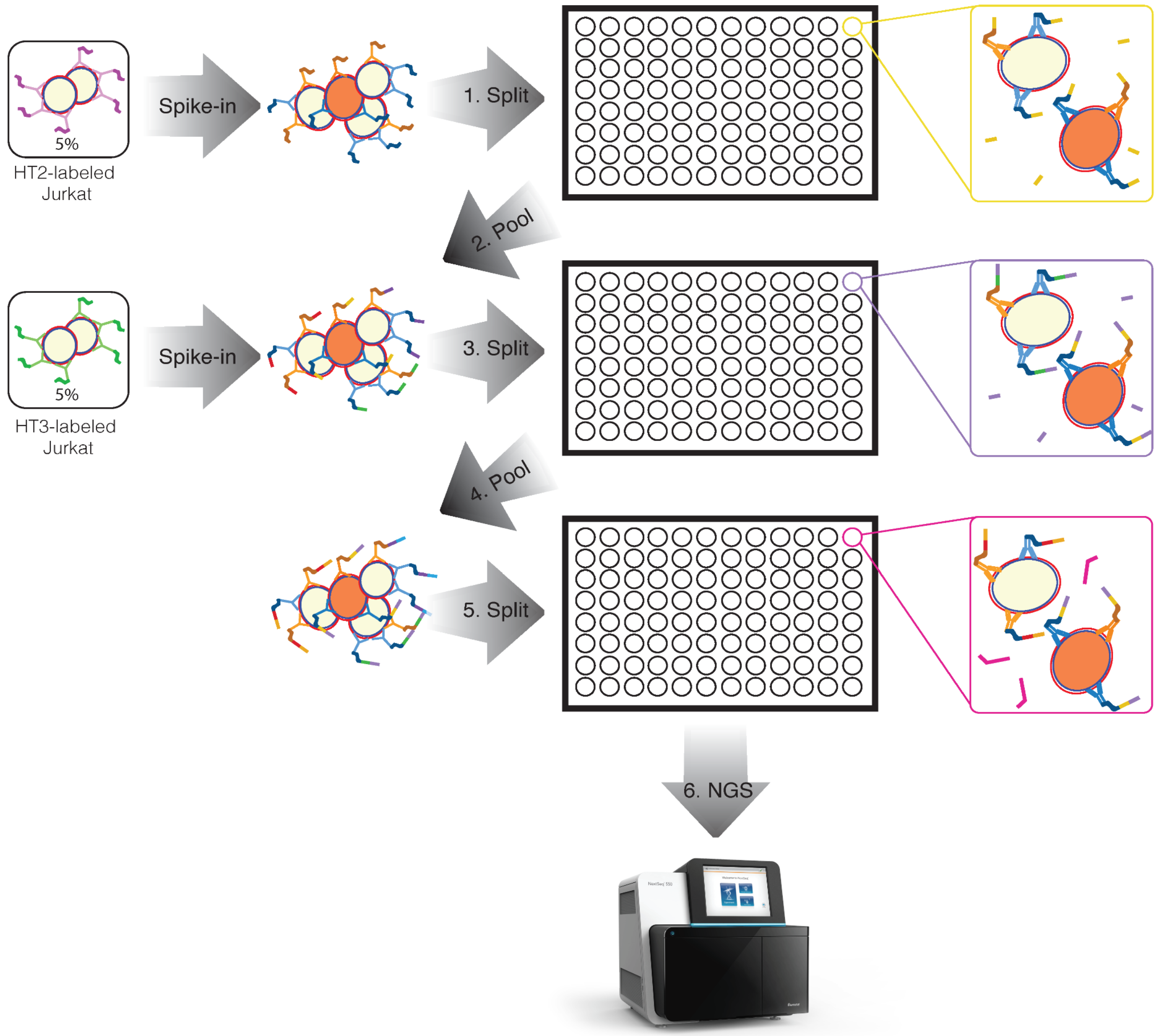

Fig. S1. Experimental schematic for two cell line mixing QBC2 experiment with doped in HT2- and HT3-labeled Jurkat cells to benchmark off-target noise and errors introduced

during sample preparation and amplification.

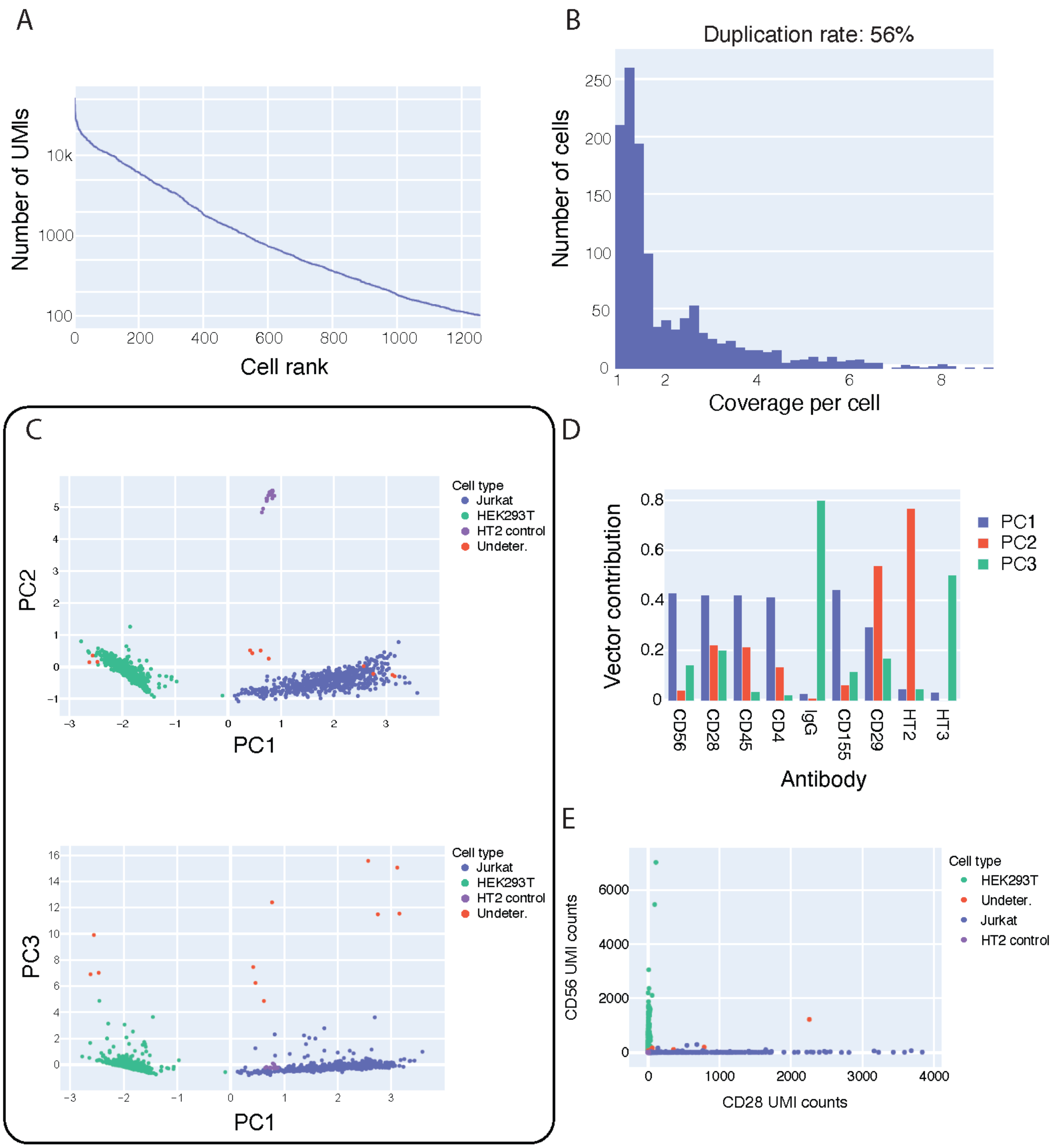

Fig. S2. Benchmarked statistics for two-cell-line experiment. (A) Unique molecular identifiers (UMIs) detected per cell, ordered by the number of UMIs detected. Cells with at least 100 unique UMIs detected were kept for further processing. (B) Average coverage per cell was calculated by taking total reads detected per cell divided by the number of unique UMIs. The duplication rate, defined as the number of UMIs detected more than a single time, is 56%. (C) Top three components of principal component analysis of two-cell-line experiment. Three populations of known cells—Jurkat,

HEK293T, HT2-stained control Jurkat cells—are distinct and clustered by k-means with k=4. A small fraction of cells show unclear characteristics, expressing both CD4 and CD56, resulting in ambiguous cell-type calling and being labeled as “undetermined”; these cells are expected to occur at low frequency and hypothesized to originate non-specific antibody binding or be an artifact of library preparation or sequencing (as interpreted from the PC3 vector in Fig. S2D). (D) Decomposition of the top three principal components. Principal component 1 features cell markers that distinguish the two main cell types (Jurkat, HEK293T). Principal component 2 is heavily dominated by HT2, serving to separate the control HT2 cells from the other two stained populations. Principle component 3 is driven mainly by signal from the IgG and HT3 markers and separates a population of cells that are not expected to exist within our mixture, indicating a noisy subset. (E) CD28 is another marker that is unique to Jurkat cells and antibody counts were quantified as an alternative way to discern the two cell lines.

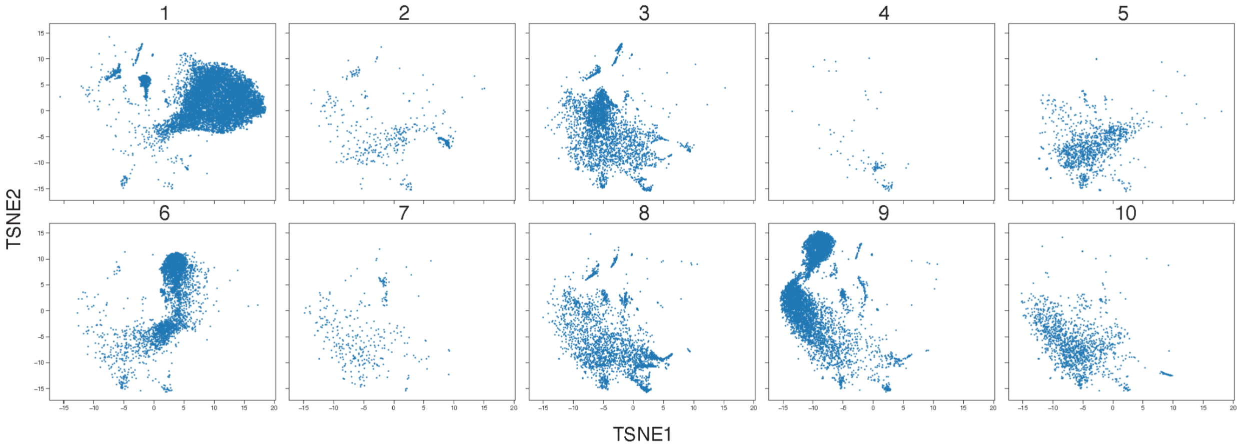

Fig. S3. Patient cells in 2D TSNE-space (as in Fig 3). Single panels are separated by patient ID as determined by hashtag labeling.

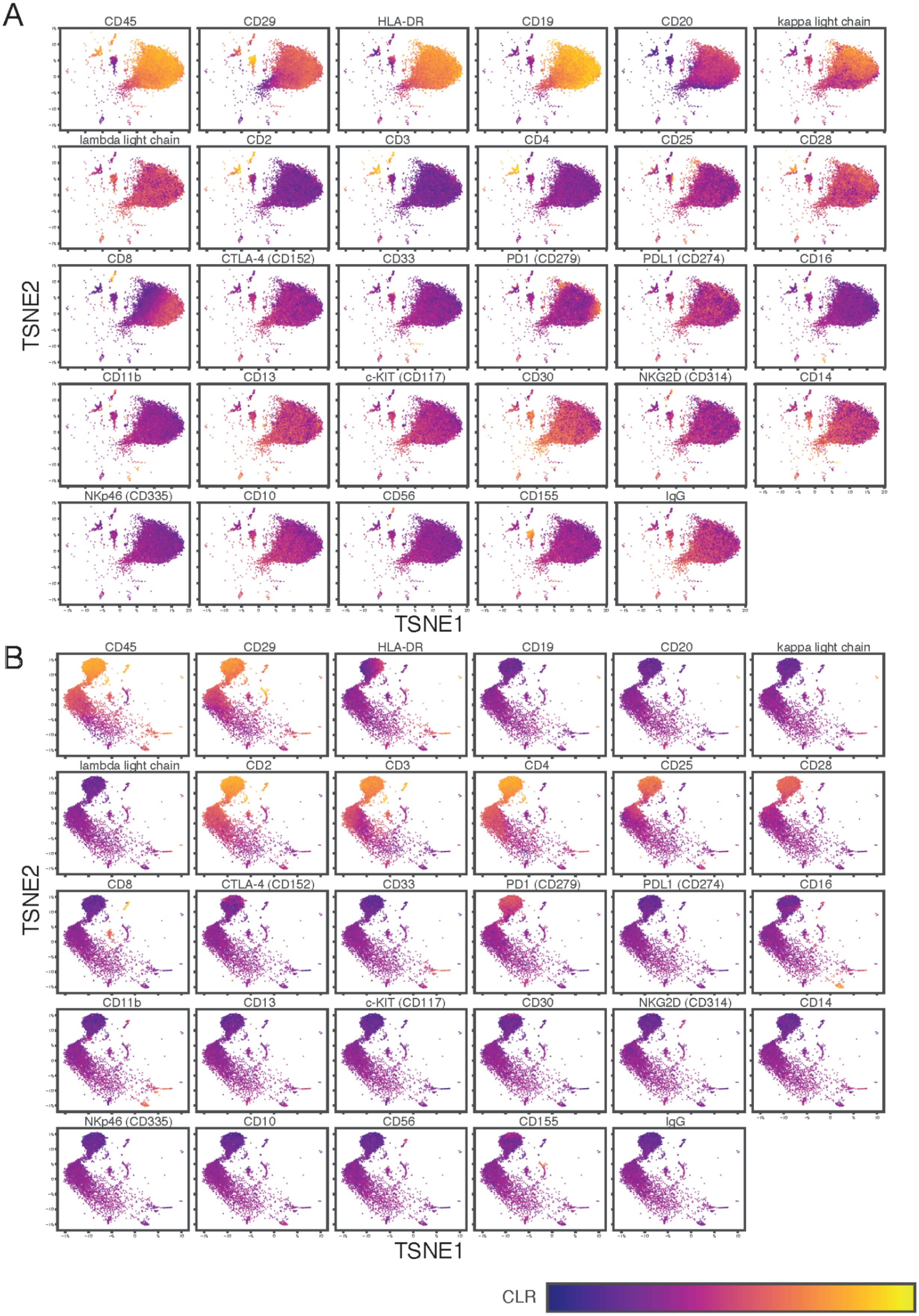
A
CD45
CD29
HLA-DR
CD19
CD20
kappa light chain
lambda light chain
CD2
CD3
CD4
CD25
CD28
CD8
CTLA-4 (CD152)
CD33
PD1 (CD279)
PDL1 (CD274)
CD16
CD11b
CD13
c-KIT (CD117)
CD30
NKG2D (CD314)
CD14
NKp46 (CD335)
CD10
CD56
CD155
IgG
TSNE2
TSNE1
B
CD45
CD29
HLA-DR
CD19
CD20
kappa light chain
lambda light chain
CD2
CD3
CD4
CD25
CD28
CD8
CTLA-4 (CD152)
CD33
PD1 (CD279)
PDL1 (CD274)
CD16
CD11b
CD13
c-KIT (CD117)
CD30
NKG2D (CD314)
CD14
NKp46 (CD335)
CD10
CD56
CD155
IgG
TSNE2
TSNE1
CLR

Fig. S4. Two dimensional TSNE of pooled patient samples, color-coded by CLR-transformed protein scores for all 29 proteins sampled. Color scales are normalized for each individual protein to show maximal dynamic range. Only cells from (A) patient 1 and (B) patient 9 are shown.
