# Supplementary material for: Quantifying protein abundance on single cells using split-pool sequencing on DNA-barcoded antibodies for diagnostic applications
Source: Sci Rep. 2022 Jan 18;12:884. doi: 10.1038/s41598-022-04842-7 (PMC8766443; doi:10.1038/s41598-022-04842-7)
Supplement: Supplementary file 4 — Supplementary Legends. [file 41598_2022_4842_MOESM4_ESM.docx]

Supplementary Table 1. Experimental reagents and cost breakdown.

Supplementary Table 2. Hashtag IDs for 10 patient samples.

Supplementary Table 3. Antibodies used, catalog numbers, and associated DNA barcodes.

Supplementary Table 4. Primers used.

Supplementary Table 5. Count matrix for patient sample experiment.

Supplementary Table 6. Plate map for SRA file names.
